# Imagery As a Core Process in the Creativity of Successful and Awarded Artists and Scientists and Its Neurobiological Correlates

**DOI:** 10.3389/fpsyg.2016.00351

**Published:** 2016-03-11

**Authors:** Rosa Aurora Chavez

**Affiliations:** ^1^Washington International Center for CreativityWashington, DC, USA; ^2^Department of Psychiatry and Behavioral Health, George Washington UniversityWashington, DC, USA; ^3^Instituto Nacional de Psiquiatría Dr. Ramón de la Fuente MuñizMexico City, Mexico

**Keywords:** creativity, imagery, creative cognition, phenomenology, neurobiology of creativity

## Abstract

This perspective paper presents an integration of neuroimaging and phenomenological data obtained in a sample that included highly creative, internationally awarded scientists and/or artists. The cerebral blood flow was evaluated during the performance of standardized creativity tasks from the Torrance Tests of Creative Thinking Verbal Form. The phenomenological data comprised both, their experiences and processes related to their creative careers and their experiences during the performance of the creative thinking tasks during the acquisition of the brain imaging data. Highly creative individuals presented a significantly higher activation of areas involved in motor imagery and described that their creative process is frequently triggered by the spontaneous and often surprising emergence of what is being named here as *primordial imagery*: a sudden, multimodal, multiintegrative, highly condensed representation that is germinative, unleashing insight and multiple associations and possibilities for meaning. As evidenced in creativity, imagery is a process through which we perceive our own minds, allowing us further symbolization and access to our thoughts, possibly facilitating neural pathways.

In the upcoming section I will briefly describe the study that followed up and our results, which have been published elsewhere (Chávez-Eakle et al., 2007). Afterward I will present phenomenological data that was also gathered but has not been previously published. In the last section I will discuss these results aiming for understanding the role of imagery in creativity, proposing new directions.

## Cerebral Blood Flow (CBF) and Creative Thinking

Twelve individuals were invited to participate in the study, the sample was recruited from a cohort of 100 participants and included eminent, internationally awarded, artists and/or scientists in the peak of their production. In addition to their actual highly creative performance, their creative potential had been assessed using the TTCT Figural form B. These psychometric tests have been used to evaluate divergent thinking (Torrance and Safter, 1999), have shown high reliability and high predictive validity (Torrance, 1988, 1990, 1993) and their structure and scoring categories have been the template for multiple subsequent tests developed in the field of creativity. The TTCT provide a creativity index (CI) and also scores for the following dimensions of the creative process: fluency, originality, and flexibility for the verbal form; and fluency, originality, elaboration, resistance to premature closure, and abstractness of titles for the figural form. In the latter, a cluster of additional points are scored by the presence of other creative strengths such as emotional expressiveness, story telling articulateness, movement or action, expressiveness of titles, synthesis of incomplete figures, unusual visualization, internal visualization, extending or breaking boundaries, humor, richness of imagery, colorfulness of imagery, and fantasy. The CI obtained with the figural TTCT was used as the parameter for the selection of the participants. Group I was integrated by individuals with a CI equal or greater than 139 (above percentile 95). Group II was composed of individuals with CI = 103–111, the middle of the normal distribution. cerebral blood flow (CBF) imaging was performed using SPECT. Two TTCT verbal-A tasks: “Just Suppose” as a warming-up activity, and “Unusual Uses” administered immediately after the injection of the radiotracer. The purpose of this study, as it was designed, was to compare the CBF between individuals with outstanding vs. average creative performance. As it has been described in a previous publication (Chávez-Eakle et al., 2007) subjects with a high creative performance showed significantly greater CBF activity in right precentral gyrus, right culmen, left, and right middle frontal gyrus, right frontal rectal gyrus, left frontal orbital gyrus, and left inferior gyrus (BA 6, 10, 11, 47, 20), and cerebellum; confirming bilateral cerebral contribution. These structures have been involved in cognition, emotion, working memory, imagery, and novelty response; suggesting an integration of perceptual, volitional, cognitive, and emotional processes in creativity. For more details please see the original source (Chávez-Eakle et al., 2007). Some of the areas presenting greater CBF in highly creative individuals correspond to what has been described as the Default Mode Network (DMN), particularly medial prefrontal cortex, the dorsomedial subsystem, and the medial temporal lobe. DMN activation has been associated to daydreaming, mind wandering, envisioning future (Cole and Schneider, 2007; Buckner et al., 2008), cognitive flexibility and conceptual shifts (Vatansever et al., 2015). Interestingly, at the same time, some activated areas correspond to what has been described as the Cognitive Control Network (CCN), particularly dorsolateral prefrontal cortex and parietal structures (Alexopolus et al., 2012) which could be related to the intentionality also involved in creativity. The cerebellum and the cortical structures involved in working memory are activated as well, which could suggest that these networks are facilitated.

## The Role of Imagery

Although the mentioned verbal tasks of the TTCT are not regarded as imagery tasks, they rely on it. For the first task (“Just Suppose”) the participants were asked to imagine a given improbable situation, and all the things that would happen as a consequence if that situation became real. For the second task (“Unusual Uses”) they had to think about all the possible different uses for cardboard boxes, and how would they transform these objects. Those individuals with higher creativity scores showed significantly grater activation of the right precentral gyrus (BA 6) premotor and supplementary motor cortex, and left middle frontal gyrus (BA 6), and area that integrates cognition and emotion, affect and meaning; these areas participate in the assimilation of sensory information, modulate the impulses transmitted to primary motor areas, and are involved in the learning of new motor programs, motor imagery (Parsons et al., 1995; Malouin et al., 2003), and auditory imagery (Zatorre et al., 1996). Increased activity in BA 6 has been observed even when subjects are only imagining complex movements in hands and fingers (Rhawn, 1996), phantom-limb movements (Roux et al., 2003), and during sexual arousal (Mouras et al., 2003). High creative performance, and in particular creative fluency correlated to a higher activity in the left parietal cortex (BA 40), a multimodal assimilation area that is also involved in imagery. This area was found to be bigger in Albert Einstein’s brain (Afifi and Bergman, 2005), a highly creative individual whose vivid imagery (e.g., seeing himself riding a rocket at light speed) led him to the elaboration of his theories. Einstein’s expanded parietal regions were related to his preference for thinking in sensory impressions, including visual images rather than words (Falk, 2009).

There were not significant differences in the activation of primary visual cortex between the groups, which could be related to the facts that: (a) They all visualized images, (b) They did not focus on the detail of those images, they focused on their transformations. When a task requires imagining high resolution detail it is more likely to observe activation of the primary visual cortex (Kosslyn and Thompson, 2003; Kosslyn et al., 2010), but when a task requires spatial judgment -which is mediated by the parietal lobe- activation of the primary cortex is less likely (Kosslyn and Thompson, 2003; Kosslyn et al., 2010). (c) We used SPECT. The more sensitive the neuroimaging technique, the more likely to observe activation of the primary visual cortex (Kosslyn et al., 2001; Kosslyn et al., 2010).

However, there were significant differences in the activation of areas related to motor imagery (what they imagine themselves doing). These participants were imagining themselves manipulating and transforming the materials they were required to visualize. They imagined themselves performing actions, building and moving, which involves kinaesthetic perceptual representations.

## Phenomenological Data

The phenomenological data that was gathered before, during and after the brain imaging acquisition has not been published before. These data comprise the phenomenological description of their own creative process, and the phenomenological description of their experience answering the TTCT verbal tasks during the SPECT.

Phenomenological Descriptions of their Creative Process: The participants were interviewed using an open ended questionnaire *ad hoc* that started with the question: “Describe the moment when you felt the most Creative.” Highly creative individuals reported vivid imagery as a key component of their creative process, regardless the domain. Artists, writers, composers, and even scientist often developed their works from the sudden presentation of imagery, which will be named here as a *primordial imagery:* a sudden, multimodal, multiintegrative, highly condensed representation that is germinative, unleashing insight and multiple associations and possibilities for meaning. Followed by further engagement, evoking more detail or related images, sensations, sounds, smells, and/or actions, triggering new associations. Their creative process at early stages involved creating a narrative to represent and/or understand these imagery contents. For instance, a writer described how an image popped up in his head almost like a dream while being awake, and from there he began to wonder what else. Imagining and describing. Some composers had visual representations within their minds and used musical sounds as the medium to narrate these images. Others had sudden auditory primordial imagery and they continued threading sounds from there. One painter said that painting from life was just the training to be able to paint internal images that popped in her mind like sudden visions. She often completed the painting it in her mind before attempting to represent it in the canvas, nonetheless, representation always fails and the actual painting is always a bridge, a compromise between the primordial imagery and what can be represented. Perhaps the following description will be striking, since it was communicated by a molecular biologist: *I was in the train. Coming into a tunnel. And there was a curve. Looking at how the train was coming into the tunnel and the light around it brought to my mind the clear image of the receptors binding. And I was able to see the mechanism that I had not been able to decipher before. I was seeing my self in the lab with results. I wrote then the main part of the paper and the discussion. I just went to the lab to prove it. I became famous for that, but I didn’t think the editors would have allowed me to describe this process in the methodology; how I had first envisioned the results vividly in my mind.*

**FIGURE 1:**
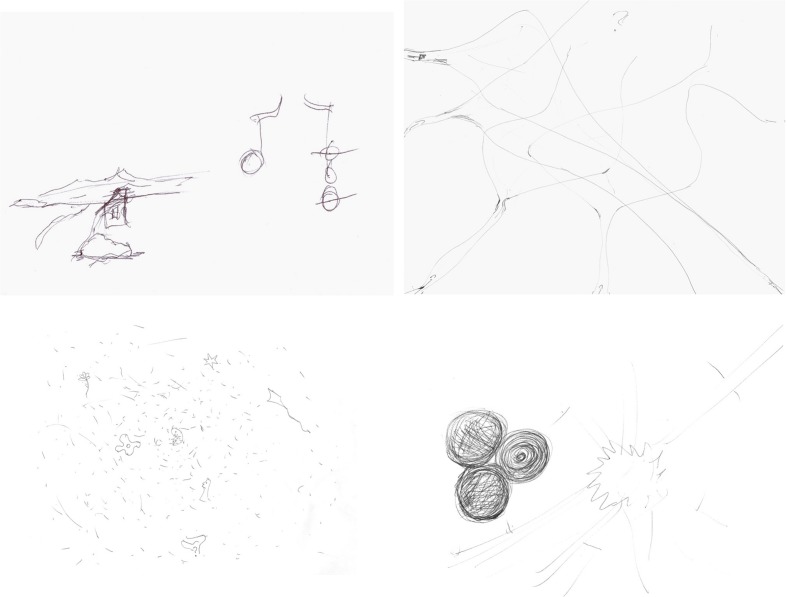
**Representations of the Creative Process, Creativity Index percentile 99**.

**FIGURE 2:**
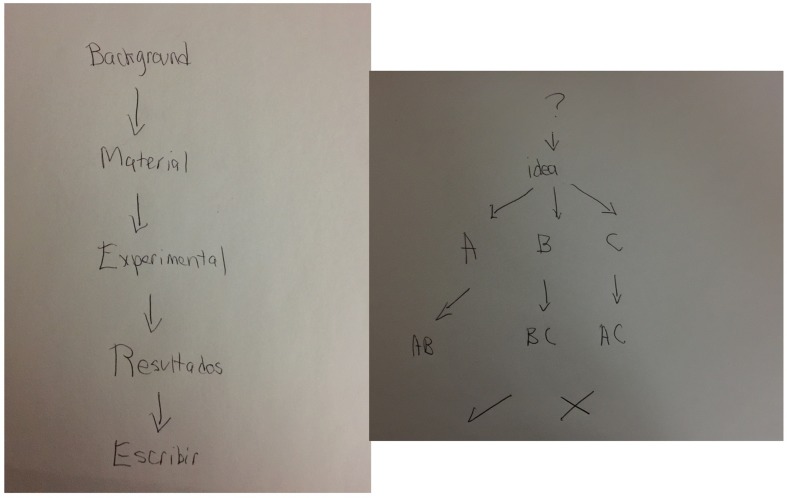
**Representations of the Creative Process, Creativity Index percentile 50**.

At the end of the questionnaire, all the participants were required to think about an image that represented their creative process. They could write and/or draw. Every verbal response was audio recorded. Researchers have been puzzled by individual differences in the intensity or quality of imagery. Creativity has a normal distribution that could be the case of imagery as well. Highly creative participants developed predominantly visual representations (**Figure 1**). One scientist draws a volcano and said: *the volcano is the image, and the image becomes words. A fire in the brain. Everything starts with a sensation that finds its own words to be described.* A composer and film maker draw a whirlpool saying: *my work is the spin within the whirlpool, the space is the mind in blank, and there’s suddenly a rhythm, and the hands and the mind find each other again*. A painter draw three ascending lines saying: *it is a match, an ignited moment, holly pyrotechnics!* A scientist draw his own head open and a flow chart emerging, the first share contained the label: image, sound, idea, through an arrow it led to the label memory, another arrow to discipline, knowledge, technique, and the final arrow led to something tangible. Another painter draw a labyrinth of neurons with narrow and open spaces, saying: *through narrow spaces, feelings, and images collide at high speed*. Another scientists said while she was drawing: *first it is the white page, all possibility and all uncertainty, then millions of dots, images colliding faster than I can develop. Suddenly the agglutinate gives birth to shapes: a face, a comet, a star, an embryo, in a fast an spontaneous movement as if they popped out by themselves, then I search for meaning*. A sculptor drew himself inside of a sac and said: *this is me within the cosmos perceiving it from within, the internal world communicates through sudden symbols*. A scientist just wrote: it’s pop corn! Interestingly, those individuals with creative performance on the 50 percentile tended to draw diagrams (**Figure 2**), words, and arrows such as: identify the problem, resources, experiment, results, statements, writing. Another example: problem, idea, combinations of ideas. Another one just wrote: follow the scientific method.

Phenomenological description of their experience answering the TTCT during the SPECT: those individuals scoring higher on the TCCT also described a more vivid experience of their imagined scenarios, as if they were seeing, hearing, touching, and smelling with the eyes of the mind. Experiencing this imagery often provoked their heart beat to go faster. They also spontaneously described feeling “high” happier, energized by the experience of coming up with ideas.

## Discussion

Combining both, neuroimaging data with phenomenological descriptions allows us to have a more panoramic view of how imagery is a core process in creativity. It also allows us to challenge traditional views. The imagery displayed by highly creative individuals seems to go beyond the conceptualization of imagery as the retrieval of memories of perception (Guillot and Collet, 2010) and provides further evidence to other approaches that consider that imagery does not necessarily result only from the recall of previous perception but it can also be created by combining and modifying stored perceptual information in novel ways (Kosslyn et al., 2001). One of the key requisites for creativity is novelty. Sometimes having as result making a leap beyond what was thinkable in a given era, allowing us to expand conceptual fields. According to the data obtained in this sample, the more imagery diverges and goes beyond stored perceptual information the more novelty results. Furthermore, a breakthrough corresponded to imagery that was apparently not related to stored perceptual information; its newness was experienced as striking.

Since creativity has such evolutionary relevance and the production of primordial imagery seems to trigger the creative process, imagery could be the immediate way we perceive our own minds, in a condensed, polysemic way. Imagery engages brain mechanisms that are used in perception and action (Kosslyn et al., 2010) and mechanisms that control physiological processes such as heart rate and breathing (Guillot and Collet, 2010), having effects much like those that occur with the corresponding perceptual stimuli. Imagery is an integrative process involving pathways of memory, emotion, perception, and action. The participants described how imagery allowed them to achieve more complex responses over a given time which suggests that imagery could facilitate neural pathways. Evidence in this direction has been described in the literature where imagery has been found to enhance performance and memory (Kosslyn et al., 2001), and skill acquisition allowing the development of a mental blueprint (Holmes et al., 2010). In addition, several techniques of facilitation used in the field of creativity to potentiate or to unlock creative processes (e.g., Creative Problem Solving; the Incubation Model of Teaching; Future Problem Solving, among others) rely on imagery to trigger associations. They require a state of free floating attention where judgment is deferred in order to continue associating. A state that promotes both imagery and free association.

## Conclusion

The creative process is frequently triggered by the spontaneous and often surprising emergence of what is being named here as *primordial imagery*: a sudden, multimodal, multiintegrative, highly condensed representation that is germinative unleashing insight and multiple associations and possibilities for meaning. As evidenced in creativity, imagery is a process through which we perceive our own minds, allowing us further symbolization and access to our thoughts, possibly facilitating neural pathways.

## Ethics Statement

All the procedures were performed in compliance with the relevant laws and institutional guidelines and were approved by the National Institute of Psychiatry “Ramón de la Fuente” (INPRF) Ethics and Scientific Committees. Informed consent was obtained and signed by all the subjects.

## Author Contributions

The author confirms being the sole contributor of this work and approved it for publication.

## Funding

This study was funded by the National Institute of Psychiatry Ramon de la Fuente.

## Conflict of Interest Statement

The author declares that the research was conducted in the absence of any commercial or financial relationships that could be construed as a potential conflict of interest.

## References

[B1] Afifi A. K. Bergman R. A. (2005). *Functional Neuroanatomy: Text and Atlas*, 2nd Edn New York, NY: McGraw-Hill.

[B2] Alexopolus G. S. Hoptman M. J. Kanellopoulos D. Murphy C. F. Lim K. O. Gunning F. M. (2012). Functional connectivity in the cognitive control network and the default control network in late life depression. *J. Affect. Disord.* 139 56–65. 10.1016/j.jad.2011.12.002 22425432 PMC3340472

[B3] Buckner R. L. Andrews-Hanna J. R. Schacter D. L. (2008). The brain’s default network: anatomy, function, and relevance to disease. *Ann. N. Y. Acad. Sci.* 1124 1–38. 10.1196/annals.1440.011 18400922

[B4] Chávez-Eakle R. A. Graff-Guerrero A. García-Reyna J. C. Vaugier V. Cruz-Fuentes C. (2007). Cerebral blood flow associated with creative performance: a comparative study. *Neuroimage* 38 519–528. 10.1016/j.neuroimage.2007.07.059 17884587

[B5] Cole M. W. Schneider W. (2007). The cognitive control network: integrated cortical regions with dissociable functions. *Neuroimage* 37 343–360. 10.1016/j.neuroimage.2007.03.071 17553704

[B6] Falk D. (2009). New information about Albert Einstein’s brain. *Front. Evol. Neurosci.* 1:3 10.3389/neuro.18.003.2009 PMC2704009 19597545

[B7] Guillot A. Collet C. (2010). *The Neurophysiological Foundations of Mental and Motor Imagery.* Oxford: Oxford University Press.

[B8] Holmes P. Cumming J. Edwards M. (2010). “Movement imagery, observation and skill,” in *The Neurophysiological Foundations of Mental and Motor Imagery*, eds Guillot A. Collet C. (Oxford: Oxford University Press), 254–269.

[B9] Kosslyn S. M. Thompson W. L. (2003). When is visual cortex activated during visual mental imagery? *Psychol. Bull.* 129 723–776. 10.1037/0033-2909.129.5.723 12956541

[B10] Kosslyn S. M. Thompson W. L. Ganis G. (2001). Neural foundations of imagery. *Nat. Rev. Neurosci.* 2 635–642. 10.1038/35090055 11533731

[B11] Kosslyn S. M. Thompson W. L. Ganis G. (2010). “Multimodal Images in the Brain,” in *The Neurophysiological Foundations of Mental and Motor Imagery*, eds Guillot A. Collet C. (Oxford: Oxford University Press), 3–16.

[B12] Malouin F. Richards C. L. Jackson P. L. Dumas F. Doyon J. (2003). Brain activations during motor imagery of locomotor-related tasks: a PET study. *Hum. Brain Mapp.* 19 47–62. 10.1016/j.cortex.2014.09.022 12731103 PMC6872050

[B13] Mouras H. Stoléru S. Bittoun J. Glutron D. Pélégrini-Issac M. Paradis A. L. (2003). Brain processing of visual sexual stimuli in healthy men: a functional magnetic resonance imaging study. *Neuroimage* 20 855–869. 10.1016/S1053-8119(03)00408-7 14568457

[B14] Parsons L. M. Fox P. T. Downs J. H. Glass T. Hirsch T. B. Martin C. C. (1995). Use of implicit motor imagery for visual shape discrimination as revealed by PET. *Nature* 375 54–58. 10.1038/375054a0 7723842

[B15] Rhawn J. (1996). *Neuropsychiatry, Neuropsychology, and Clinical Neuroscience*, 2nd Edn Baltimore, MD: Williams & Wilkins.

[B16] Roux F. E. Lotterie J. A. Cassol E. Lazorthes Y. Sol J. C. Berry I. (2003). Cortical areas involved in virtual movement of phantom limbs: comparison with normal subjects. *Neurosurgery* 53 1342–1352. 10.1227/01.NEU.0000093424.71086.8F 14633300

[B17] Torrance E. P. (1988). “The nature of creativity as manifest in its testing,” in *The Nature of Creativity*, ed. Sternberg R. J. (New York, NY: Cambridge University Press), 43–75.

[B18] Torrance E. P. (1990). *Torrance Tests of Creative Thinking.* Bensenville, IL: Scholastic Testing Service.

[B19] Torrance E. P. (1993). The beyonders in a thirty year longitudinal study of creative achievement. *Roeper Rev.* 15 131–135. 10.1080/02783199309553486

[B20] Torrance E. P. Safter H. T. (1999). *Making the Creative Leap Beyond. Creative Education.* Buffalo, NY: Foundation Press.

[B21] Vatansever D. Menon D. K. Manktelow A. E. Sahakian B. J. (2015). Default mode dynamics for global functioning integration. *J. Neurosci.* 35 15254–15262. 10.1523/JNEUROSCI.2135-15.2015 26586814 PMC4649001

[B22] Zatorre R. J. Halpern A. R. Perry D. W. Meyer E. Evans A. C. (1996). Hearing in the mind’s ear: a PET investigation of musical imagery and perception. *J. Cogn. Neurosci.* 8 29–46. 10.1162/jocn.1996.8.1.29 23972234

